# Knowledge of cardiovascular diseases and associated risk factors in the general adult population of Jeddah, Saudi Arabia: A cross-sectional study examining gender disparities

**DOI:** 10.1097/MD.0000000000038566

**Published:** 2024-06-14

**Authors:** Ranya Alawy Ghamri

**Affiliations:** a Family Medicine Department, Faculty of Medicine, King Abdulaziz University, Jeddah, Saudi Arabia.

**Keywords:** cardiovascular disease, educational programs, gender differences, knowledge, prevention

## Abstract

To assess knowledge about cardiovascular diseases (CVD) among the general population, we emphasized gender-related disparities and other lifestyle and health-related factors. This cross-sectional study was conducted among 395 individuals from the general population of Jeddah, Saudi Arabia. An online questionnaire was administered to assess knowledge of CVD types, symptoms, and risk factors. The majority of participants identified coronary heart disease (73.7%) as having CVD, with no sex effect. Males had higher knowledge regarding cerebrovascular disease (44% vs 31.5%) and congenital heart diseases (60% vs 51.9%) as being part of CVD, while they had lower knowledge of peripheral arterial disease (44% vs 50.7%) than females, respectively (*P* < .05). Males exhibited better knowledge of heart attack and stroke symptoms than females. Knowledge was optimal for major CVD risk factors, such as smoking (90.6%) and high low-density lipoprotein cholesterol levels (85.1%); however, gaps were observed in recognizing diabetes (56.2%) and stress (69.4%) as factors for CVD. The mean overall knowledge score was 16.33 ± 5.72 25, with no difference between sexes (*P* = .239). Predictors of good CVD knowledge included university-level education, daily healthy food consumption, and perceived life as highly stressful; nonetheless, sex showed no significant effect. While the general population displayed a suboptimal understanding of CVD, notable sex disparities were observed, highlighting the need for tailored public health interventions. Emphasizing cognitive and behavioral aspects can foster better prevention and management strategies, given the evident gender disparities.

## 1. Introduction

Cardiovascular diseases (CVD) are a set of heart and blood vessel disorders, including coronary heart disease (CHD), cerebrovascular disease, peripheral arterial disease, congenital heart disease, rheumatic heart disease, deep vein thrombosis, and pulmonary embolism.^[1]^ CVD is one of the most debilitating diseases and is associated with high morbidity and mortality rates. According to the World Health Organization, CVD is the main cause of mortality worldwide, accounting for 31% of all fatalities.^[2,3]^ In Saudi Arabia, CVD is estimated to account for nearly half of all the deaths (42%).^[2,3]^ Moreover, according to a systematic review using Gulf Region data, which included Saudi Arabia among other countries, the prevalence of CVD was found to be higher than that in developed countries such as some European countries and other developing countries such as India.^[3]^ This observation aligns with the escalating global trend of detrimental health behaviors and other CVD risk factors. A recent study from Saudi Arabia involving a representative sample of 2047 individuals revealed striking prevalence rates of several cardiovascular risk factors, including low physical activity (69.4%), obesity (49.6%), unhealthy diet (34.4%), dyslipidemia (32.1%), hypertension (30.3%), and diabetes (25.1%). Current smoking, a known risk factor for CVD, was observed in 12.2% of participants.^[4]^

Based on these observations, effective prevention strategies and education programs should be implemented in Gulf countries to reverse rising CVD-induced mortality and morbidity. It has been observed that a low public knowledge and awareness about CVD and related risk factors is associated with inadequate control of the resulting health consequences.^[5]^ It is crucial to note that most CVD factors are controllable or modifiable, making CVD one of the most avoidable causes of mortality.^[6]^ Therefore, efficient strategies should include promoting people’s understanding of CVD and its risk factors to improve health-promoting attitudes, behaviors, and lifestyles and ultimately reduce the burden of CVD in the general population. This implies appraising knowledge about CVD and its related risk factors to design more targeted education programs.

Among the various factors that shape the burden of CVD, sex is a crucial determinant that influences various other determinants of cardiovascular health. It is noteworthy that males and females experience distinct impacts from CVD, possibly due to biological, psychosociological, and behavioral differences. Consequently, while males exhibit a higher risk of CVD development, females tend to bear a greater burden of CVD-related morbidity and mortality.^[7]^ Moreover, lifestyles related to cardiovascular health, such as nutrition, physical activity, and smoking, are likely to be strongly dependent on sex.^[8–10]^ Consequently, these sex differences may have important implications in prevention strategies, particularly when compounded by cognitive disparities in awareness and knowledge of CVD and its associated risk factors. Therefore, a critical, yet relatively unexplored question arises: does the understanding of CVD and its risk factors vary between sexes? Our current knowledge database offers limited insights, particularly in the context of Saudi Arabia.

In an attempt to fill this gap, the primary objective of this study was to assess the knowledge levels concerning CVD and its associated risk factors among a population from Jeddah, Saudi Arabia. Additionally, we aimed to evaluate the influence of sex on knowledge and prevention determinants, seeking to understand how these disparities might affect the effectiveness of preventive approaches. Lastly, this study aimed to elucidate the other demographic, lifestyle, and health-related factors that correlate with CVD awareness and knowledge among the participants. The findings of this study will provide crucial data for the development and implementation of future public health policies and strategies. Furthermore, the results could guide the tailoring of these programs to effectively reach and serve the population most vulnerable to CVD.

## 2. Methods

### 2.1. Design and setting

This population-based cross-sectional study was conducted in Jeddah, Saudi Arabia, between January and August 2021.

### 2.2. Participants and sampling

This study targeted the general adult population of Jeddah, focusing on individuals aged ≥18 years. Using an online source (https://www.surveysystem.com/sscalc.htm), the sample size was calculated to detect an unknown proportion of participants with adequate knowledge about CVD, with a 95% confidence interval, 80% statistical power, and 0.05 type 1 error. The sample size was 390 participants. Adult residents of Jeddah aged ≥18 years were eligible to participate. Healthcare professionals, medical students, and those residing outside the Jeddah region were excluded from the study.

### 2.3. Data collection

Data for this study were collected using a structured, self-administered online questionnaire disseminated via Google Forms across various social media platforms. The questionnaire was adopted from a study by Awad and Al-Nafisi.,^[11]^ and where applicable, items were rephrased to fit the target population’s context. The questionnaire’s version used for this study was formulated in plain Arabic and was designed to cover the following domains:

− Demographic and lifestyle data: This section captured essential demographic details such as age, nationality, and education level, as well as lifestyle and health-related factors including self-perceived health and weight status, smoking habits, exercise routine, dietary habits, stress levels, and immediate family history of CVD.
− Health-related data: This section primarily focused on obtaining information about any preexisting chronic diseases (such as hypertension and diabetes mellitus), current medication intake for these conditions, recent measurements of blood pressure, cholesterol, fasting blood glucose, and the last time these health parameters, including body weight, were checked.
− Knowledge about CVD: This section aimed to evaluate participants’ awareness and understanding of various types of CVD and their ability to recognize the symptoms of heart attacks and strokes. For each listed CVD and potential symptom, participants were provided with 3 response options: “Yes,” “No,” or “I do not know.” This format enabled the assessment of participants’ knowledge while also allowing for uncertainty.
− Knowledge about risk factors of CVD: Participants were queried about their knowledge of several risk factors associated with CVD, such as smoking, unhealthy diet, physical inactivity, obesity, stress, family history of CVD, high low-density lipoprotein cholesterol levels, hypertension, and diabetes. Respondents could select from the 3 response options: “Yes,” “No,” or “I do not know,” permitting an honest evaluation of their understanding while accommodating any indecisiveness.

The questionnaire was validated for face and content by 2 independent physicians, 1 public health specialist, and 1 family physician, as well as 1 research methodologist. Subsequently, it was pretested on a small group of 10 participants to ensure clarity and relevance.

The duration of completion of the questionnaire was approximately 10 minutes. Participants were informed of the purpose of the study before participation, and informed consent was obtained online.

### 2.4. Ethical clearance

Ethical approval for this research was obtained from the Research Ethics Committee of King Abdul-Aziz University, Jeddah, Saudi Arabia, under Reference No 182-21. The study was conducted in compliance with the ethical guidelines and standards set forth by the committee. Additionally, all participants were informed about the purpose of the study and their rights, and informed consent was explicitly obtained from the study questionnaire. Confidentiality of the participants’ data was maintained rigorously, with no personal identifiers being collected. The study design also ensured that no vulnerable groups were unduly targeted, and the exclusion of healthcare professionals and medical students aimed to prevent potential bias in knowledge assessment.

### 2.5. Statistical methods

Data were statistically analyzed using the Statistical Package for Social Sciences version 26. Qualitative data were expressed as numbers and percentages, and the chi-square test (*χ*^2^) was used to test the relationship between variables. Quantitative data are expressed as mean and standard deviation (mean ± SD), and the Mann–Whitney test was used to analyze nonparametric variables. Statistical significance was set to indicate statistical significance. The questionnaire’s internal consistency was analyzed by calculating Cronbach alpha, which was found to be 0.897 indicating that the knowledge scale is reliable and suitable for score calculation.

## 3. Results

### 3.1. Participants’ characteristics and lifestyle habits

A total of 395 participants responded to the interview, with a mean (SD) age of 37.29 ± 13.92 years. The majority of respondents were female (68.4%), Saudi (96.7%), married (56.2%), and held university degrees (64.1%). More than half (51.4%) were employed, and the most common self-perceived health status was “excellent” (47.1%). A significant proportion (41.3%) perceived themselves as overweight, whereas 78.5% were identified as nonsmokers. Most participants did not engage in regular exercise (69.6% exercised 0-2 times a week) and did not consume healthy food every day (82.8%). With regard to stress, the majority felt that their lives were “relatively stressful” (58%). Notably, 35.4% of the close relatives had a family history of CVD (Table 1).

**Table 1:** Sociodemographic and lifestyle characteristics (N = 395).

Parameter	Level	Statistics (n, %)
Age	Mean ± SD	37.29 ± 13.92
Body mass index	Mean ± SD	26.8 ± 6.04
Gender	Male	125 (31.6)
	Female	270 (68.4)
Nationality	Non-Saudi	13 (3.3)
	Saudi	382 (96.7)
Marital status	Widowed	9 (2.3)
	Divorced	26 (6.6)
	Married	222 (56.2)
	Single	138 (34.9)
Educational level	University	253 (64.1)
	Diploma	38 (9.6)
	High school	70 (17.7)
	Less than high school	8 (2)
	Master’s degree or PhD	2 (6.6)
Employment	Employed	203 (51.4)
	Retired	49 (12.4)
	Student	73 (18.5)
	Unemployed	70 (17.7)
Monthly income	5000–10,000 SAR	(27.6) 109
	<5000 SAR	(34.4) 136
	>10,000 SAR	(38) 150
Self-perceived health status	Poor	2 (0.5)
	Fair	15 (3.8)
	Good	44 (11.1)
	Very good	148 (37.5)
	Excellent	186 (47.1)
Self-perceived weight	Underweight	24 (6.1)
	Normal	168 (42.5)
	Overweight	163 (41.3)
	Obese	40 (10.1)
Smoking status	Nonsmoker	310 (78.5)
	Ex-smoker	24 (6.1)
	Current smoker	61 (15.4)
If you are a past smoker, when did you stop smoking? (n = 24)	In the last 6–12 mo	(8.4) 2
	<6 mo ago	(37.5) 9
	>12 mo ago	13 (54.1)
Weekly exercise frequency†	0–2 times	(69.6) 275
	3–5 times	(22.3) 88
	≥5 times	(8.1) 32
Frequency of healthy food intake‡	Everyday	(17.2) 68
	Not everyday	(82.8) 327
Self-perceived lifestyle stress†	Free from stress	(9.6) 38
	Relatively stressful	(58) 229
	Stressful	(21.3) 84
	Very stressful	(11.1) 44
CVD among close relatives§	No	255 (64.6)
	Yes	140 (35.4)

Results are frequencies (percentages), except if otherwise specified.

CVD = cardiovascular disease, SAR = Saudi riyal.

† Participants answered the following question: In a typical week, how many days do you practice at least 30 minutes of exercise (such as walking, running, cycling, and jogging)?

‡ Participants answered the following question: How often do you eat healthy food? (Plenty of fruits and vegetables, foods that are low in saturated fat, cholesterol, and salt and high in fiber).

§ Include mother, father, sister, brother, and/or own child.

Gender comparisons showed that significantly more females (90.4%) perceived their health as very good or excellent than males (72.0%, *P* < .001). However, in terms of health behaviors, fewer females reported regular exercise (25.5% vs 40.8%, *P* = .007) and daily consumption of healthy food (14.1% vs 24.0%, *P* = .015) than their male counterparts. The prevalence of current smokers was significantly higher among males (36.8% vs 5.6%, *P* < .001) (results not depicted in tables).

### 3.2. Health-related data and cardiovascular morbidity

The self-reported frequencies of chronic diseases found hypertension (15.4%), diabetes mellitus (11.1%), high blood cholesterol (14.9%), as the most frequent. When evaluating recent health metrics, 7.8% had high blood pressure, 10.1% had elevated blood cholesterol levels, and 7.6% had elevated fasting blood glucose levels. As for health checkups, the majority had their blood pressure checked within the last 1 to 3 months (42.3%), but a notable 15.2% never had it checked. Cholesterol checks within the last 1 to 3 months were reported by 22.5% of the participants, although 27.1% had never done so. Similarly, fasting blood glucose checks were most recent within the last 1 to 3 months for 27.6% of participants, yet 24.8% had never had it checked. When evaluating body weight, most participants (53.4%) had checked within the last 1 to 3 months (Table 2).

**Table 2:** Health-related data (N = 395).

Parameter	Level	n (%)
Do you have any of the following chronic diseases?	Hypertension	61 (15.4)
	Diabetes mellitus	44 (11.1)
	High blood cholesterol	59 (14.9)
	Congenital heart disease	15 (3.8)
Medications	For hypertension	50 (12.7)
	For diabetes mellitus	43 (10.9)
	For high blood cholesterol	(7.3) 29
	For congenital heart disease	13 (3.3)
Recent blood pressure	High	31 (7.8)
	I do not know	120 (30.4)
	Normal	244 (61.8)
Recent blood cholesterol	High	40 (10.1)
	I do not know	184 (64.6)
	Normal	171 (43.3)
Recent fasting blood glucose	High	30 (7.6)
	I do not know	152 (38.5)
	Normal	213 (53.9)
Last time blood pressure checked	>1 yr ago	43 (10.9)
	Within the last 1–3 mo	167 (42.3)
	Within the last 7–12 mo	57 (14.4)
	Never checked before	60 (15.2)
	Unsure/I do not know	68 (17.2)
Last time blood cholesterol checked	>1 yr ago	52 (13.2)
	Within the last 1–3 mo	89 (22.5)
	Within the last 7–12 mo	58 (14.7)
	Never checked before	107 (27.1)
	Unsure/I do not know	89 (22.5)
Last time blood glucose checked	>1 yr ago	45 (11.4)
	Within the last 1–3 mo	109 (27.6)
	Within the last 7–12 mo	53 (13.4)
	Never checked before	98 (24.8)
	Unsure/I do not know	90 (22.8)
Last time body weight checked	>1 yr ago	34 (8.6)
	Within the last 1–3 mo	211 (53.4)
	Within the last 7–12 mo	38 (9.6)
	Never checked before	51 (12.9)
	Unsure/I do not know	61 (15.4)

Gender comparisons showed that the prevalence of hypertension (*P* < .001), diabetes (*P* < .001), hypercholesterolemia (*P* = .011), and CHD (*P* < .001) was higher among male participants; accordingly, higher percentages of males were taking medications for these diseases than females (*P* < .001 for all). Men also had higher abnormal values of recently measured blood pressure (*P* < .001) and fasting blood glucose (*P* < .001) (results not presented in tables).

### 3.3. Knowledge about cardiovascular diseases

Most participants recognized CHD (73.7%) as CVD. Recognition of other CVDs varied, with 59.2% correctly identifying rheumatic heart disease, 54.4% for congenital heart diseases, 48.6% for peripheral arterial diseases, 44.3% for deep vein thrombosis and pulmonary embolism, and 35.4% for cerebrovascular disease. A notable percentage of respondents indicated “I do not know” for many of these diseases (approximately one-third), demonstrating gaps in awareness. Regarding heart attack symptoms, the majority of participants recognized chest pain or discomfort (78.5%) and difficulty in breathing or shortness of breath (81%) as indicators of a heart attack. On the other hand, awareness of stroke symptoms was relatively varied: 64.3% of participants associated sudden dizziness, trouble walking, or loss of balance or coordination with a stroke; 60.5% recognized sudden confusion or trouble speaking; and 60% experienced sudden numbness or weakness in the face, arm, or leg with a stroke (Table 3).

**Table 3:** Knowledge about types of cardiovascular disease and symptoms of heart attack and stroke, in total population and female versus male participants (N = 395).

Dimension/knowledge level	Total, N = 395 n (%)	Female, N = 270 n (%)	Male, N = 125 n (%)	*χ* ^2^	*P* value
Types of cardiovascular diseases					
Coronary heart disease					
I do not know	98 (24.8)	71 (26.3)	27 (21.6)		
No	6 (1.5)	5 (1.9)	1 (0.8)		
Yes	291 (73.7)	194 (71.9)	97 (77.6)	1.76	.141
Cerebrovascular disease					
I do not know	148 (37.5)	102 (37.8)	46 (36.8)		
No	107 (27.1)	83 (30.7)	24 (19.2)		
Yes	140 (35.4)	85 (31.5)	55 (44)	8	**.018** *
Peripheral arterial diseases					
I do not know	147 (37.2)	103 (38.1)	44 (35.2)		
No	56 (14.2)	30 (11.1)	26 (20.8)		
Yes	192 (48.6)	137 (50.7)	55 (44.0)	6.64	**.036** *
Rheumatic heart disease					
I do not know	128 (32.4)	90 (33.3)	38 (30.4)		
No	33 (8.4)	17 (6.3)	16 (12.8)		
Yes	234 (59.2)	163 (60.4)	71 (56.8)	4.73	.094
Congenital heart diseases					
I do not know	134 (33.9)	96 (35.6)	38 (30.4)		
No	46 (11.6)	34 (12.6)	12 (9.6)		
Yes	215 (54.4)	140 (51.9)	75 (60.0)	2.36	**.030** *
Deep vein thrombosis and pulmonary embolism					
I do not know	145 (36.7)	104 (38.5)	41 (32.8)		
No	75 (19)	50 (18.5)	25 (20)		
Yes	175 (44.3)	116 (43.0)	59 (47.2)	1.2	.547
Symptoms of a heart attack?					
Pain or discomfort in the jaw, neck, or back					
I do not know	152 (38.5)	111 (41.1)	41 (32.8)		
No	106 (26.8)	88 (32.6)	18 (14.4)		
Yes	137 (34.7)	71 (26.3)	66 (52.8)	29.37	**<.001** *
Feeling weak, lightheaded, or faint					
I do not know	88 (22.3)	60 (22.2)	28 (22.4)		
No	57 (14.4)	41 (15.2)	16 (12.8)		
Yes	250 (63.3)	169 (62.6)	81 (64.8)	0.4	.817
Chest pain or discomfort					
I do not know	60 (15.2)	40 (14.8)	20 (16.0)		
No	25 (6.3)	21 (7.8)	4 (3.2)		
Yes	310 (78.5)	209 (77.4)	101 (80.8)	3.03	.219
Pain or discomfort in the arms or shoulder					
I do not know	108 (27.3)	73 (27.0)	35 (28.0)		
No	51 (12.9)	35 (13.0)	16 (12.8)		
Yes	236 (59.7)	162 (60.0)	74 (59.2)	0.04	.980
Difficulty in breathing or shortness of breath					
I do not know	59 (14.9)	34 (12.6)	25 (20)		
No	16 (4.1)	14 (5.2)	2 (1.6)		
Yes	320 (81)	(82.2) 222	98 (78.4)	6	.050
Symptoms of stroke					
Sudden numbness or weakness in the face, arm, or leg					
I do not know	125 (31.6)	85 (31.5)	40 (32.0)		
No	33 (8.4)	27 (10.0)	6 (4.8)		
Yes	237 (60)	158 (58.5)	79 (63.2)	3.08	.214
Sudden confusion or trouble speaking or understanding others					
I do not know	114 (28.9)	75 (27.8)	39 (31.2)		
No	42 (10.6)	26 (9.6)	16 (12.8)		
Yes	239 (60.5)	169 (62.6)	70 (56.0)	1.76	.413
Sudden trouble seeing with 1 or both eyes					
I do not know	132 (33.4)	86 (31.9)	46 (36.8)		
No	46 (11.6)	30 (11.1)	16 (12.8)		
Yes	217 (54.9)	154 (57.0)	63 (50.4)	1.52	.468
Sudden dizziness, trouble walking, or loss of balance or coordination					
I do not know	113 (28.6)	79 (29.3)	34 (27.2)		
No	28 (7.1)	21 (7.8)	7 (5.6)		
Yes	254 (64.3)	170 (63.0)	84 (67.2)	0.93	.626
Severe headache of unknown cause					
I do not know	139 (35.2)	103 (38.1)	36 (28.8)		
No	60 (15.2)	53 (19.6)	7 (5.6)		
Yes	196 (49.6)	114 (42.2)	82 (65.6)	22.6	**<.001** *

* Statistically significant difference (*P* < .50).

When comparing gender, a higher percentage of males were aware that cerebrovascular disease (44.0% vs 31.5%; *P* = .018) and congenital heart diseases (60.0% vs 51.9%; *P* = .030) were part of CVD, with reference to females, respectively. Conversely, a higher percentage of females acknowledged peripheral arterial disease as a CVD (50.7% vs 44.0%; *P* = .036) than males. Regarding the recognition of heart attack and stroke symptoms, 2 notable disparities were observed between the sexes. First, males more frequently identified “pain or discomfort in the jaw, neck, or back” as a symptom of a heart attack (52.8%) compared to females (26.3%), with a significance of *P* < .001. Second, “severe headache of unknown cause” was more commonly recognized by males (65.6%) than by females (42.2%), with a significance of *P* < .001. However, for other aspects concerning the types of CVD and related symptoms, the knowledge between the 2 sexes showed no significant difference, as detailed in Table 3.

### 3.4. Knowledge about cardiovascular disease risk factors

The participants displayed a strong understanding of the risk factors associated with CVD. The vast majority (82.3% to 90.6%) recognized smoking, high low-density lipoprotein cholesterol levels, positive family history of CVD, obesity, low physical inactivity, and unhealthy dietary habits as risk factors. However, areas of relative uncertainty included the associations of diabetes (56.2%) and stress (69.4%) with CVD. Overall, when considering all aspects of CVD knowledge, participants achieved a mean score of 16.33 out of a possible 25, suggesting that there is still room for improvement in CVD awareness (Table 4).

**Table 4:** Knowledge about risk factors of cardiovascular diseases, in total population and female versus male participants (N = 395).

Risk factor/knowledge level	Total, N = 395 n (%)	Female, N = 270 n (%)	Male, N = 125 n (%)	*χ* ^2^	*P* value
Smoking					
I do not know	29 (7.3)	13 (4.8)	16 (12.8)		
No	8 (2)	7 (2.6)	1 (0.8)		
Yes	358 (90.6)	250 (92.6)	108 (86.4)	9.13	**.010** *
Unhealthy dietary habits such as diets high in saturated fats, cholesterol, and salt					
I do not know	47 (11.9)	30 (11.1)	17 (13.6)		
No	23 (5.8)	18 (6.7)	5 (4.0)		
Yes	325 (82.3)	222 (82.2)	103 (82.4)	1.48	.475
Low physical activity					
I do not know	39 (9.9)	23 (8.5)	16 (12.8)		
No	29 (7.3)	25 (9.3)	4 (3.2)		
Yes	327 (82.8)	222 (82.2)	105 (84.0)	5.89	.053
Obesity					
I do not know	47 (11.9)	32 (11.9)	15 (12.0)		
No	12 (3)	9 (3.3)	3 (2.4)		
Yes	336 (85.1)	229 (84.8)	107 (85.6)	0.25	.881
Stress					
I do not know	74 (18.7)	49 (18.1)	25 (20.0)		
No	47 (11.9)	35 (13.0)	12 (9.6)		
Yes	274 (69.4)	186 (68.9)	88 (70.4)	0.99	.608
Positive family history of cardiovascular disease					
I do not know	47 (11.9)	28 (10.4)	19 (15.2)		
No	18 (4.6)	16 (5.9)	2 (1.6)		
Yes	330 (83.5)	226 (83.7)	104 (83.2)	5.18	.075
High LDL cholesterol levels					
I do not know	49 (12.4)	30 (11.1)	19 (15.2)		
No	10 (2.5)	9 (3.3)	1 (0.8)		
Yes	336 (85.1)	231 (85.6)	105 (84.0)	3.34	.188
Hypertension					
I do not know	67 (17)	45 (16.7)	22 (17.6)		
No	25 (6.3)	21 (7.8)	4 (3.2)		
Yes	303 (76.7)	204 (75.6)	99 (79.2)	3.02	.221
Diabetes					
I do not know	101 (25.6)	72 (26.7)	29 (23.2)		
No	72 (18.2)	61 (22.6)	11 (8.8)		
Yes	222 (56.2)	137 (50.7)	85 (68.0)	13.84	**.001** *

LDL = low-density lipids.

* Statistically significant difference (*P* < .50).

Sex comparison showed that a significantly higher percentage of females (92.6%) than males (86.4%) were knowledgeable that smoking was a risk factor for CVD (*P* = .010). In contrast, a significantly higher percentage of males (68.0%) than females (50.7%) were aware that diabetes constitutes a risk factor for CVD (*P* = .001). No other significant sex differences were observed with regard to knowledge of other risk factors (Table 4).

### 3.5. Knowledge scores

In the total study population of 395 participants, the mean (SD) CVD types knowledge score (out of a possible 6) was 3.15 ± 1.95, while the mean (SD) knowledge score about CVD risk factors was 7.11 ± 2.27 out of a possible 9. The participants displayed a mean (SD) understanding of heart attack symptoms, scoring 3.17 ± 1.53 out of 5, which was significantly higher among males than females (*P* = .015). For stroke symptoms, the mean score was slightly lower at 2.89 ± 1.81 5. Aggregating these findings, the overall CVD knowledge score (from a total possible score of 25) was 16.33 ± 5.72 (Table 5).

**Table 5:** Comparative CVD knowledge scores by gender in total population.

Knowledge dimension	Total (mean ± SD)	Female (mean ± SD)	Male (mean ± SD)	Student *t* test statistics (*P* value)
CVD types (total score = 6)	3.15 ± 1.95	3.09 ± 1.89	3.29 ± 2.08	0.92 (.353)
CVD risk factors (total score = 9)	7.11 ± 2.27	7.06 ± 2.22	7.23 ± 2.37	1.45 (.145)
Heart attack symptoms (total score = 5)	3.17 ± 1.53	3.08 ± 1.43	3.36 ± 1.72	2.42 (.015*)
Stroke symptoms (total score = 5)	2.89 ± 1.81	2.83 ± 1.78	3.02 ± 1.88	1.17 (.239)
Overall CVD knowledge score (total = 25)	16.33 ± 5.72	16.07 ± 5.35	16.91 ± 6.43	1.85 (.064)

CVD = cardiovascular disease.

* Statistically significant difference (*P* < .50).

### 3.6. Predictors of cardiovascular disease knowledge

Multivariate linear regression analysis examining the independent predictors of knowledge (overall knowledge score) revealed that having university-level education (B = 0.67, *P* = .045), consuming healthy food daily (B = 0.30; *P* = .007), and perceived life as very stressful (B = 1.18; *P* = .027) were independent predictors of a higher overall knowledge score regarding CVD among participants. No sex effect was noted in the multivariate analysis (Table 6).

**Table 6:** Multivariate logistic regression analysis of factors associated with knowledge about cardiovascular disease (N = 395).

Variable	B	Wald	*P* value	Odds ratio (95% CI)
Sex	0.03	0.008	.927	1.03 (0.53–1.99)
Age	0.55	2.22	1.36	0.57 (0.27–1.19)
Nationality	1.44	1.65	.199	4.24 (0.46–38.58)
Education	0.67	4.03	**.045** *	0.5 (0.26–0.98)
Monthly income	0.25	0.41	.52	1.29 (0.59–2.81)
Frequency of physical activity (minimum duration, 30 min)	0.46	0.6	.437	0.62 (0.19–202)
Eating healthy food	0.30	7.34	**.007** *	0.27 (0.1–0.69)
Family history of CVD	0.55	3.4	.065	0.57 (0.31–1.03)
Smoking status	0.55	0.89	.345	0.57 (0.15–1.8)
Perception of lifestyle as being very stressful	1.18	4.86	**.027** *	0.3 (0.1–0.87)

CVD = cardiovascular disease.

* Statistically significant difference (*P* < .50).

## 4. Discussion

### 4.1. Summary of findings

A population well aware of CVD and its risk factors is more likely to engage in preventive behaviors, promptly recognize symptoms, and seek timely medical interventions. Conversely, gaps in knowledge or misconceptions can lead to delays in diagnosis, suboptimal management, and increased morbidity. This study sought to assess the levels of knowledge about CVD among the general population, with an emphasis on elucidating the nuances presented by sex differences and other determinants’ awareness of CVD. In this study involving 395 participants with an average age of 37.29 years, several observations on cardiovascular health, lifestyle habits, and knowledge were made. Remarkably, males presented a higher prevalence of chronic conditions and cardiovascular risk factors, while they were more prone to engage in regular physical activity and daily consumption of healthy food, compared to females. Overall, participants had adequate awareness of CHD as a CVD, but knowledge concerning other CVDs and their symptoms was less adequate. Gender differences in knowledge were significant, with males typically having better awareness of specific diseases and symptoms than females. However, some gaps were observed; notably, the participants had a less clear understanding of the connection between stress, diabetes, and CVD. The mean overall CVD knowledge score was 16.33 out of 25, which can be considered moderate assuming a theoretical arithmetic mean of 12.5 (25/2). Independent factors associated with better CVD knowledge included higher education, daily healthy food intake, and viewing life as stressful.

### 4.2. Public knowledge about cardiovascular disease types

Public awareness of CVD types and their warning signs is key to primary prevention, disease detection, and morbidity reduction. However, insufficient knowledge about CVDs and their risk factors can impede effective prevention and treatment. Therefore, recognizing and addressing individuals’ beliefs and attitudes is essential for guiding behavioral shifts. We observed a relatively high level of knowledge about CHD, as the majority of participants identified it as CVD. Moreover, most respondents recognized the symptoms of heart attack. However, there was inadequate knowledge about other types of CVD, and a considerable proportion of the respondents did not recognize stroke symptoms. Similarly, a cross-sectional study conducted by Awad et al involving 900 respondents revealed that CHD was the most frequently identified type of CVD and the majority were aware of heart attack symptoms, with chest pain and shortness of breath being the most commonly known. In the same study, almost half (47.8%) of the participants did not recognize any symptoms of stroke.^[11]^ In a systematic review of 20 studies from sub-Saharan countries, while awareness of CVD was notably high in specific groups, overall knowledge about CVDs was low. Most studies found that <50% of the respondents had an in-depth understanding of CVDs. For example, only 19% of workers in Nigerian University Hospital had comprehensive knowledge of CVDs. This suggests that despite high awareness, there is a significant knowledge gap regarding CVDs in sub-Saharan Africa.^[12]^

Various models, such as the health belief model, emphasize the pivotal role of knowledge in shaping health behaviors and ensuring sustained changes.^[13]^ These models propose that a deeper understanding of a disease condition can shape a patient’s attitude and behavior, enhance adherence to treatment, and result in decreased disease prevalence and fewer complications.^[14]^ This underscores the pressing need to strengthen community-based educational programs, as the information currently provided to the general population appears inadequate. This is particularly crucial for acute CVD conditions like stroke, where the adage “time is brain” rings true.^[15]^ Early recognition of symptoms by patients is vital for timely consultation and intervention, thereby ensuring swift and effective management.

### 4.3. Public knowledge about cardiovascular risk factors

In our study, there was notable awareness among participants about CVD risk factors, such as smoking, hypercholesterolemia, obesity, unhealthy dietary habits, and physical inactivity. However, awareness was less pronounced regarding diabetes and stress. With an overall mean knowledge score of 16.33 out of 25, these findings suggest that while Saudi adults possess a considerable understanding of the conditions predisposing them to cardiovascular events, there remains a clear need for targeted educational interventions to address the identified knowledge gaps. Awad et al observed similar findings when assessing public awareness of CVD risk factors, where smoking, obesity, unhealthy diet, and physical inactivity were correctly identified by over four-fifths of the respondents.^[11]^ Previous studies have also demonstrated that when asked about cardiovascular risk factors, over half of the participants from the general population identified physical activity, smoking, overweight, and high cholesterol.^[16,17]^ In contrast to our results, a study from Jeddah, Saudi Arabia, found that a family history of diabetes was among the most commonly reported risk factors for coronary artery disease, identified by 47.2%.^[18]^ Being aware of the modifiable risk factors of CVD positively impacts engagement in healthy behaviors and reduces harmful lifestyle habits.^[19]^ Therefore, it is crucial to improve individuals’ general culture concerning protective lifestyle and promoting behaviors of cardiovascular health.

### 4.4. Gender effect in lifestyle behaviors and knowledge about cardiovascular disease

Females tended to have lower compliance with regular exercise and a healthy diet, while smoking was more common in males. This is consistent with the observations made by previous studies from Gulf Cooperation Council countries, which demonstrated higher activity rates among men than women, likely resulting from sociocultural and environmental factors contributing to physical inactivity in females.^[20]^ While some studies suggest that women prioritize healthy eating and exhibit more concerns about unhealthy diets,^[21,22]^ recent findings show that women often struggle more than men to maintain a nutritious diet, potentially because of financial constraints and limited autonomy in household financial choices.^[23]^ Regarding smoking, there is clearly a male-dominated behavior among adults in Middle Eastern countries.^[24]^

Interestingly, while there were statistically significant gender differences in knowledge, the percentage difference between the genders was not pronounced enough to conclude an impactful difference in knowledge levels. Inconsistently, Awad et al observed higher knowledge of CVD in females compared to males.^[11]^ On the other hand, Muhihi et al indicated greater CVD knowledge among men.^[25]^ Gender differences do not solely arise from biological differences but are also deeply associated with societal norms, roles, and expectations.

Men and women have distinct experiences and exposures that influence their health behaviors and knowledge. The observed sex-related disparities in CVD morbidity can positively promote knowledge and awareness. Thus, given their higher cardiovascular morbidity, male participants might be more exposed to information about CVD through personal experience, regular doctor visits, or health interventions. This firsthand experience might boost their understanding and awareness of certain aspects of the disease, thus explaining the higher knowledge among males observed in this study. At the same time, women, traditionally seen as family caregivers, often tend to gather knowledge about diseases to care for their family members. Their role as caregivers might make them more receptive to health information, enhancing their knowledge and awareness of the broader aspects of CVD. On the other hand, gender-related disparities can negatively impact cardiovascular health awareness. Cultural perceptions of masculinity may prevent men from seeking or admitting a lack of health knowledge.^[26]^ Despite their caregiving roles, women might prioritize family health over their own, leading to personal knowledge gaps. Both genders’ behaviors and misconceptions can exacerbate these gaps, impacting their understanding of personal risk.

Therefore, it is important to understand that gender plays a dual role in changing susceptibility to CVD by being a biological determinant that may influence cardiovascular health and a sociocultural factor that shapes cardiovascular health behaviors and knowledge. In Austria, where CVD remains a major health concern, significant knowledge gaps have been observed in both sexes, but with distinct challenges. While women tend to misjudge their personal risks, they benefit from targeted education programs that enhance their awareness and preventive actions. Conversely, men generally showed lower levels of awareness and were less engaged in preventive measures. This underscores the necessity of sex-specific public health strategies to address CVD.^[27]^ Thus, taking into consideration gender disparities regarding CVD attitudes and knowledge would increase the effectiveness of health campaigns and educational programs by disseminating more personalized information. More specifically, gender-specific gaps in knowledge and healthy hygiene can be used to help address issues according to gender, notably, the importance of regular exercise and a balanced diet to women, and the cessation of smoking to males. Simultaneously, it can be used to promote peer learning, where men and women are encouraged to share their knowledge and experiences within their gender groups. Men with comorbidities and clinical experiences can be ambassadors for early CVD detection among peers, while women with caregiving experience can promote broader CVD awareness.

### 4.5. Gender-based difference in cardiovascular morbidity

In this study, the self-reported medical history showed that the frequency of CVD varied among participants, with hypertension (15.4%) being the most frequently reported and CHD (3.8%) being the least frequently reported. Given that the average age of the participants was 37.29 years, a low reporting of CVD history is expected. However, the self-reported rates suggest early onset of CVD in the Saudi population. Notably, men reported significantly higher rates of hypertension, diabetes, CHD, hypercholesterolemia, and related treatment than women. This aligns with the existing evidence that males inherently carry a greater CVD risk due to biological differences.^[28]^ Female hormones, such as estrogen, offer cardioprotective effects,^[29]^ whereas factors linked to the Y chromosome, such as heightened systemic inflammation and changes in the renin-angiotensin system, increase CVD risk.^[30]^ Understanding these gender-based distinctions in CVD prevalence can guide targeted knowledge dissemination and prevention strategies, ensuring that each sex receives appropriate and effective health interventions.

### 4.6. Education, healthy diet, and life stress are associated with better cardiovascular disease knowledge

The results of this study showed that individuals living in Saudi Arabia who had university-level education, good dietary habits, or described their lifestyle as very stressful had a higher tendency to display awareness about CVD and cardiovascular risk factors. These findings are similar to those of the study conducted by Awad et al,^[11]^ which showed that a high level of education and regular eating of a healthy diet are independent predictors of CVD knowledge. Likewise, Hassen et al observed an independent association between the level of education and CVD knowledge score (adjusted *β* = 0.11, 95% CI: 0.03–0.18) and CVD risk perception (0.23, 95% CI: 0.04–0.41), in addition to engagement in healthy behaviors including physical activity and healthy diet intentions.^[31]^ The positive correlation between healthy diet consumption and CVD knowledge seems to be a typical finding, as people who know more about the risk factors of CVD, including unhealthy foods, are likely to find more meaning in following more rigorous nutritional hygiene. At the same time, following more prudent dietary habits on a daily basis would motivate further reading about the recommended prevention strategies for common diseases, including those for CVD, ultimately enhancing the level of insight regarding how to preserve cardiovascular health. A higher educational level facilitates information acquisition and retention. Exposure to high amounts of stress in life would also encourage individuals to look for ways to improve their overall well-being.

### 4.7. Implications for interventions

The study findings imply that community education about CVD and its related risk factors should be enhanced. In particular, educational programs should focus on increasing the general population’s awareness of the hazards of an unhealthy lifestyle by consuming an unbalanced diet, practicing low levels of physical activity, and consuming different smoking products. Moreover, these programs should aim to provide sufficient knowledge about the alarming symptoms of CVD and cerebrovascular disease and the actions that should be taken when presenting one of them. Special targets of CVD awareness spreading interventions are populations with a lower educational background, those consuming unhealthy food, and those with a small perception of stress in life since these individuals seem to be more susceptible to the lack of cardiovascular health-related information. Additionally, the identified sex disparities in lifestyle habits and CVD knowledge highlight the need for tailored health interventions. For males, emphasis should be placed on smoking cessation and management of existing CVD risk factors. For females, interventions could focus on promoting regular exercise and a balanced diet. Both genders would benefit from targeted awareness campaigns focusing on areas where knowledge deficits were observed. Investing in sex-specific public health initiatives can lead to more effective disease prevention, heightened health awareness, and the empowerment of individuals to make informed health decisions.

## 5. Limitations

Our study had certain limitations that warrant attention. First, the cross-sectional design restricts our ability to infer causality among knowledge, lifestyle, and health-related factors. Second, reliance on self-assessed knowledge may introduce a subjective bias. Third, the gender imbalance in our sample—with female participants outnumbering males 2 to 1. This is probably due to more frequent use of social media for questionnaire dissemination. This constitutes a selection bias that should be accounted for in the interpretation of the findings. Finally, the geographical scope of our study, which is confined to just 1 region in Saudi Arabia, limits the generalizability of our findings. Thus, although our research provides valuable insights, these limitations should be considered when interpreting the results and drawing conclusions.

## 6. Conclusion

This study indicated a moderate level of public knowledge concerning CVD, with some important deficiencies. It also highlights the dual impact of sex in determining cardiovascular health, particularly through knowledge and the associated behaviors and attitudes. Although both sexes exhibited comparable overall levels of CVD-related awareness, men are more prone to CVD morbidity and certain risk behaviors, such as smoking, whereas women are less adherent to a healthy diet and vigorous physical activity. By understanding how gender positively and negatively compounds knowledge, health interventions can be designed more efficaciously to control the burden of CVD, ensuring a holistic and gender-sensitive approach. Furthermore, this study revealed a strong relationship between CVD knowledge and education, nutrition, and life stress perception. These findings can help to improve the efficacy of primary prevention interventions by providing more specific educational care to the general population. However, further studies are required to expand the current understanding regarding further factors explaining sex disparities in the context of CVD in Saudi Arabia.

## Acknowledgments

The author would like to thank Drs Ghiadaa Albaz, Wejdan Ali Alshehri, Wejdan Hazim Alshehri, Wasayf Almehmadi, and Sara Mannan for their invaluable contribution to data analysis.

## Author contributions

**Conceptualization:** Ranya Alawy Ghamri.

**Data curation:** Ranya Alawy Ghamri.

**Formal analysis:** Ranya Alawy Ghamri.

**Funding acquisition:** Ranya Alawy Ghamri.

**Investigation:** Ranya Alawy Ghamri.

**Methodology:** Ranya Alawy Ghamri.

**Project administration:** Ranya Alawy Ghamri.

**Resources:** Ranya Alawy Ghamri.

**Software:** Ranya Alawy Ghamri.

**Supervision:** Ranya Alawy Ghamri.

**Validation:** Ranya Alawy Ghamri.

**Visualization:** Ranya Alawy Ghamri.

**Writing – original draft:** Ranya Alawy Ghamri.

**Writing – review & editing:** Ranya Alawy Ghamri.

## References

[1] Cardiovascular diseases. 2023. Available at: https://www.who.int/health-topics/cardiovascular-diseases#tab=tab_1. [access date October 13, 2023].

[2] Non-communicable diseases country profiles 2018. 2018. Available at: https://www.who.int/publications/i/item/9789241514620. [Access date October 13, 2023].

[3] Aljefree N Ahmed F. Prevalence of cardiovascular disease and associated risk factors among adult population in the Gulf region: a systematic review. Adv Public Health. 2015;2015:1–23.

[4] Alhabib KF Batais MA Almigbal TH. Demographic, behavioral, and cardiovascular disease risk factors in the Saudi population: results from the Prospective Urban Rural Epidemiology study (PURE-Saudi). BMC Public Health. 2020;20:1213. 32770968 10.1186/s12889-020-09298-w PMC7414714

[5] Turk-Adawi K Sarrafzadegan N Fadhil I. Cardiovascular disease in the Eastern Mediterranean region: epidemiology and risk factor burden. Nat Rev Cardiol. 2018;15:106–19. 28933782 10.1038/nrcardio.2017.138

[6] Roth GA Mensah GA Johnson CO. Global burden of cardiovascular diseases and risk factors, 1990-2019: update from the GBD 2019 study. J Am Coll Cardiol. 2020;76:2982–3021. 33309175 10.1016/j.jacc.2020.11.010 PMC7755038

[7] Möller-Leimkühler AM. Gender differences in cardiovascular disease and comorbid depression. Dialogues Clin Neurosci. 2007;9:71–83. 17506227 10.31887/DCNS.2007.9.1/ammoeller PMC3181845

[8] Wardle J Haase AM Steptoe A Nillapun M Jonwutiwes K Bellisle F. Gender differences in food choice: the contribution of health beliefs and dieting. Ann Behav Med. 2004;27:107–16. 15053018 10.1207/s15324796abm2702_5

[9] Chang S-H Chang Y-Y Wu L-Y. Gender differences in lifestyle and risk factors of metabolic syndrome: do women have better health habits than men? J Clin Nurs. 2019;28:2225–34. 30786102 10.1111/jocn.14824

[10] Al-Nozha MM Al-Mazrou YY Arafah MR. Smoking in Saudi Arabia and its relation to coronary artery disease. J Saudi Heart Assoc. 2009;21:169–76. 23960568 10.1016/j.jsha.2009.06.007 PMC3727358

[11] Awad A Al-Nafisi H. Public knowledge of cardiovascular disease and its risk factors in Kuwait: a cross-sectional survey. BMC Public Health. 2014;14:1131. 25367768 10.1186/1471-2458-14-1131 PMC4237772

[12] Boateng D Wekesah F Browne JL. Knowledge and awareness of and perception towards cardiovascular disease risk in sub-Saharan Africa: a systematic review. PLoS One. 2017;12:e0189264. 29232703 10.1371/journal.pone.0189264 PMC5726714

[13] Stretcher V Rosenstock IM. The health belief model. In: Glanz Lewis FM Rimer BK (eds). Health Behavior and Health Education: Theory, Research and Practice. San Francisco: Jossey-Bass. 1997.

[14] Adedoyin RA Erhabor GE Ojo OD. Impact of patients’ knowledge, attitude and practices on hypertension on compliance with antihypertensive drugs in a resource-poor setting. TAF Prev Med Bull. 2010;9:87–92.

[15] Saver JL. Time is brain—quantified. Stroke. 2006;37:263–6. 16339467 10.1161/01.STR.0000196957.55928.ab

[16] Krupp K Wilcox ML Srinivas A Srinivas V Madhivanan P Bastida E. Cardiovascular risk factor knowledge and behaviors among low-income urban women in Mysore, India. J Cardiovasc Nurs. 2020;35:588–98. 32084082 10.1097/JCN.0000000000000657 PMC7438232

[17] Rahman DMM Akter S Zohora F. Public knowledge of cardiovascular disease and its risk factors in Tangail, Bangladesh: a cross-sectional survey. Int J Community Med Public Health. 2019;6:1838.

[18] Almalki MA AlJishi MN Khayat MA. Population awareness of coronary artery disease risk factors in Jeddah, Saudi Arabia: a cross-sectional study. Int J Gen Med. 2019;12:63–70. 30666149 10.2147/IJGM.S184732 PMC6333320

[19] Alzaman N Wartak SA Friderici J Rothberg MB. Effect of patients’ awareness of CVD risk factors on health-related behaviors. South Med J. 2013;106:606–9. 24192590 10.1097/SMJ.0000000000000013

[20] Mabry RM Reeves MM Eakin EG Owen N. Evidence of physical activity participation among men and women in the countries of the Gulf cooperation council: a review. Obes Rev. 2010;11:457–64. 19793376 10.1111/j.1467-789X.2009.00655.x

[21] Grzymisławska M Puch EA Zawada A Grzymisławski M. Do nutritional behaviors depend on biological sex and cultural gender? Adv Clin Exp Med. 2020;29:165–72. 32017478 10.17219/acem/111817

[22] Bärebring L Palmqvist M Winkvist A Augustin H. Gender differences in perceived food healthiness and food avoidance in a Swedish population-based survey: a cross sectional study. Nutr J. 2020;19:140. 33375947 10.1186/s12937-020-00659-0 PMC7772926

[23] Njuki J Eissler S Malapit H Meinzen-Dick R Bryan E Quisumbing A. A review of evidence on gender equality, women’s empowerment, and food systems. Glob Food Secur. 2022;33:100622. 38285870

[24] Kargar S Ansari-Moghaddam A. Prevalence of cigarette and waterpipe smoking and associated cancer incidence among adults in the Middle East. East Mediterr Health J. 2023;29:749–56. 37776137 10.26719/emhj.23.077

[25] Muhihi AJ Anaeli A Mpembeni RNM. Public knowledge of risk factors and warning signs for cardiovascular disease among young and middle-aged adults in rural Tanzania. BMC Public Health. 2020;20:1832. 33256688 10.1186/s12889-020-09956-z PMC7708242

[26] Silvestrini M Chen JA. “It’s a sign of weakness”: masculinity and help-seeking behaviors among male veterans accessing posttraumatic stress disorder care. Psychol Trauma. 2023;15:665–71. 36201833 10.1037/tra0001382 PMC11107421

[27] Haidinger T Zweimüller M Stütz L Demir D Kaider A Strametz-Juranek J. Effect of gender on awareness of cardiovascular risk factors, preventive action taken, and barriers to cardiovascular health in a group of Austrian subjects. Gend Med. 2012;9:94–102. 22440721 10.1016/j.genm.2012.02.001

[28] Walli-Attaei M Joseph P Rosengren A. Variations between women and men in risk factors, treatments, cardiovascular disease incidence, and death in 27 high-income, middle-income, and low-income countries (PURE): a prospective cohort study. Lancet. 2020;396:97–109. 32445693 10.1016/S0140-6736(20)30543-2

[29] Iorga A Cunningham CM Moazeni S Ruffenach G Umar S Eghbali M. The protective role of estrogen and estrogen receptors in cardiovascular disease and the controversial use of estrogen therapy. Biol Sex Differ. 2017;8:33. 29065927 10.1186/s13293-017-0152-8 PMC5655818

[30] Khan SI Andrews KL Jennings GL Sampson AK Chin-Dusting JPF. Y chromosome, hypertension and cardiovascular disease: is inflammation the answer? Int J Mol Sci. 2019;20:2892. 31200567 10.3390/ijms20122892 PMC6627840

[31] Hassen HY Bowyer M Gibson L Abrams S Bastiaens H. Level of cardiovascular disease knowledge, risk perception and intention towards healthy lifestyle and socioeconomic disparities among adults in vulnerable communities of Belgium and England. BMC Public Health. 2022;22:197. 35093056 10.1186/s12889-022-12608-z PMC8800212

